# Up-to-date data on Pelvic Inflammatory Disease

**DOI:** 10.1590/0037-8682-0419-2021

**Published:** 2021-11-12

**Authors:** Ricardo F Savaris

**Affiliations:** 1 Universidade Federal do Rio Grande do Sul, Departamento de Ginecologia e Obstetrícia, Porto Alegre, RS, Brasil.

**Dear Dr. Correia Filho,**

(Editor-in-chief of JBSTM)

It was with great interest that I read the article published by Menezes et al. on pelvic inflammatory disease, in a recent publication of the JBSTM^1^, which was a review of the Clinical Protocol and Therapeutic Guidelines for Comprehensive Care for People with Sexually Transmitted Infections, published by the Health Surveillance Secretariat of the Brazilian Ministry of Health. Although it has been reported that the protocol was developed through the selection and analysis of available evidence, followed by discussions with specialists, I would like to share some comments about the "new" evidence provided in the 2020 Brazilian Guidelines.

Pelvic inflammatory disease (PID) is a challenging condition because it has mild or non-specific signs and symptoms^2^. The sequelae of PID can be infertility, ectopic pregnancy, and chronic pelvic pain. For these reasons, the current diagnosis of PID, proposed by the Centers of Disease Control and Prevention (CDC)^3^, has replaced the outdated criteria proposed by Hager et al. in 1983^4^ and last seen in the CDC 1998 Guidelines for Treatment of STDs^5^. Unfortunately, recent publications from the Brazilian scientific society and the Brazilian government still use the outdated 3 major + 1 minor criteria^1^ ^),(^ ^6^, despite citing an article with the new CDC criteria^2^. These "new" CDC criteria for PID can be traced back to 2002^7^. For clarification, the CDC states: "Presumptive treatment for PID should be initiated in sexually active young women and other women at risk for STDs if they are experiencing pelvic or lower abdominal pain, if no cause for the illness other than PID can be identified, and if one or more of the following minimum clinical criteria are present on pelvic examination: cervical motion tenderness **or** uterine tenderness **or** adnexal tenderness^3^. An updated version of these guidelines was released in 2021, and the diagnostic criteria did not change

The Gainesville Stage, mentioned in their article, is an outdated concept suggested by Dr. Gilles Monif in 1982, which has little meaning nowadays, since its purpose was to guide treatment, either as outpatient or inpatient, to preserve fallopian tube structure^8^. This concept was disproved in the PEACH Trial^9^. 

The treatment topic is another aspect that requires attention. Preconized treatments do not reflect up-to-date information. Our last Cochrane review on this topic was published in 2020^10^, and this was not mentioned.

In addition, the use of gentamicin divided into two or three doses per day has been replaced by one dose per day to reduce renal injury, a fact that has been consolidated in the literature. Extended-interval aminoglycoside has efficacy comparable with traditional intermittent administration; however, it offers three potential advantages: 1) the possibility of decreased nephrotoxicity (based on data from animal models), 2) ease of administration and serum concentration monitoring, and 3) reductions in administration and monitoring-related costs. Extended-interval dosing of aminoglycosides takes advantage of two pharmacodynamic properties: the post-antibiotic effect and concentration-dependent killing^11^. 

Finally, the recommendation for removing an intrauterine device (IUD) only after two doses of antibiotics has no scientific basis. Few trials were performed where the IUD was removed before treatment, and it was found that early removal of the IUD did not influence the course of the disease, although one trial had a longer duration of treatment^12^.

I hope this letter will help clinicians and policy makers on this topic.
